# Energy Storage and Thermostability of Li_3_VO_4_-Coated LiNi_0.8_Co_0.1_Mn_0.1_O_2_ as Cathode Materials for Lithium Ion Batteries

**DOI:** 10.3389/fchem.2018.00546

**Published:** 2018-11-08

**Authors:** Liubin Song, Fuli Tang, Zhongliang Xiao, Zhong Cao, Huali Zhu

**Affiliations:** ^1^Hunan Provincial Key Laboratory of Materials Protection for Electric Power and Transportation, School of Chemistry and Biological Engineering, Changsha University of Science and Technology, Changsha, China; ^2^School of Materials Science and Engineering, Changsha University of Science and Technology, Changsha, China

**Keywords:** LiNi_0.8_Co_0.1_Mn_0.1_O_2_, cathode material, electrochemical calorimetry, thermo-electrochemistry performance, thermostability, energy storage

## Abstract

The electrochemical performances and thermostability of LiNi_0._8Co_0.1_Mn_0.1_O_2_ is affected by temperature. High ambient temperature or irregular heat distribution accelerates the decline of LiNi_0.8_Co_0.1_Mn_0.1_O_2_ performance, shortens cathode material life. In this work, the energy storage and thermostability of the Li_3_VO_4_-coated LiNi_0.8_Co_0.1_Mn_0.1_O_2_ cathode material were studied for the first time by electrochemical calorimetry methode at different temperatures and rates. Results show that Li_3_VO_4_-coated LiNi_0.8_Co_0.1_Mn_0.1_O_2_ cathode material has excellent rate and cycle performance. The thermal electrochemical experiments further show that the thermal stability of Li_3_VO_4_-coated LiNi_0.8_Co_0.1_Mn_0.1_O_2_ cathode material in charge-discharge energy storage and conversion system is better than LiNi_0.8_Co_0.1_Mn_0.1_O_2_ at 30, 40, and 50°C. The enhanced performance can be attributed to the fact that Li_3_VO_4_ coating promotes the transmission of lithium ions and protects the active material from electrolyte corrosion at different temperature, as well as reduces side reaction, electrode polarization and heat generation of cathode materials. The Li_3_VO_4_-coated LiNi_0.8_Co_0.1_Mn_0.1_O_2_ cathode material has excellent energy storage properties and thermostability, which are beneficial to the development of electronic equipment.

## Introduction

As the problem of energy storage and security becomes more and more serious, the research on new energy materials becomes more and more urgent. New energy materials play a vital role in the sustainable development of human society. As the main or auxiliary power source of new energy vehicles, lithium-ion batteries cathode materials have become an indispensable part of the development of new energy vehicles (Anseán et al., 2013; Pan et al., 2018). However, the high-temperature safety of high-capacity and high-power lithium-ion batteries for automobiles has attracted considerable attention with the widespread application of lithium-ion batteries in electric vehicles (Konishi et al., 2013). Considering the increase in energy density of electric passenger power batteries, LiNi_0.8_Co_0.1_Mn_0.1_O_2_ has a high specific capacity and is expected to become the mainstream of power energy batteries. However, high nickel in the LiNi_0.8_Co_0.1_Mn_0.1_O_2_ cathode material causes several difficulties, such as thermal instability, low diffusion coefficient of lithium ions and severe capacity attenuation (Li et al., 2006; Xiong et al., 2013; Nitta et al., 2015). Currently, the commonly used means to solve the above problems are surface modification (Cho and Cho, 2010; Xiong et al., 2012; Lu et al., 2014; Zheng et al., 2015), ion doping (Luo et al., 2010; Du et al., 2015; Chen et al., 2017), core shell and gradient structure construction (Wen et al., 2015; Liao et al., 2016), and electrolyte optimisation of electrolyte (Plichta and Behl, 2000). Surface modification is the most common means used in Li_3_VO_4_ modification its ion migration skeleton structure can promote lithium ion transport. Surface modification has a broad application prospect in lithium-ion battery cathode materials. Huang et al. (2014); Zhang et al. (2017), and Wang et al. (2015) used vanadate compounds to modify the surface of LiNi_0.8_Co_0.1_Mn_0.1_O_2_. They showed that the modified LiNi_0.8_Co_0.1_Mn_0.1_O_2_ formed on the surface of cathode material with a stable interfacial film, lithium ion diffusion and improved electronic transmission and the reduced charge transfer resistance remarkably improved interfacial electrochemistry reaction activity. Although the electrochemical performance of lithium-ion batteries by Li_3_VO_4_ modification has remarkably improved, many safety problems are still observed on lithium-ion batteries for vehicle power supply, especially the high temperature of batteries, which limit their wide application in electronic equipment and electric vehicles (Anseán et al., 2016; Liu et al., 2016). Therefore, the thermo-electrochemistry and high-temperature energy storage properties of LiNi_0.8_Co_0.1_Mn_0.1_O_2_ cathode materials modified by Li_3_VO_4_ should be investigated.

The thermo-electrochemical method is a combination of electrochemical, thermodynamic, physical, and chemical methods, which is used to analyse the battery charge and discharge performance and corresponding heat production at different temperatures (Song et al., 2013). This method can not only study the electrochemical performance of the battery under different charging and discharging states, but also obtain the data of current, heat flow and voltage change over time through the LAND battery test system and isothermal calorimeter, and obtain the thermo-electrochemical parameters, providing new theoretical basis for solving the battery safety problems. Currently, researchers have extensively investigated the electrochemical performance of surface-modified lithium-ion batteries. However, the research on thermo-electrochemical and high-temperature performance should be improved. In recent years, the thermal stability, reaction kinetics and apparent activation energy of LiNi_0.8_Co_0.1_Mn_0.1_O_2_ and other cathode materials have been analyzed by differential thermal analysis, thermogravimetric analysis, and differential scanning calorimetry. However, these analyses failed to reflect the electrochemical properties at the same time (Lee et al., 2009; Fu et al., 2014; Peng and Jiang, 2016). Traditionally, the performance of electrode materials is evaluated by using charge and discharge capacity, cycle performance and rate performance, which cannot reflect their heating characteristics and high-temperature energy storage performance. Local and global studies have been conducted to couple the battery charge and discharge test device and calorimeter to investigate the temperature change or heat generation of batteries during charge and discharge cycles at different current densities (Ye et al., 2012; Xiao et al., 2014; Mcturk et al., 2018; Song et al., 2018). Eddahech et al. (2013) used electrochemical-calorimetry to evaluate the thermal effects of high-capacity commercial nickel–cobalt–manganese–lithium batteries and verified the feasibility of electrochemical-calorimetry in the thermal electrochemical study of lithium-ion batteries. Du et al. (2017) analyzed the LiFePO_4_ battery evolving law of irreversible heat production and its different rates and granular component of thermal analysis to provide effective guidance for the battery thermal management system design. Krause et al. (2012), Vallverdu et al. (2016), and Balasundaram et al. (2016) assessed the thermal behavior and electrochemical properties of lithium-ion batteries under different conditions by using a calorimeter combined with a battery charging and discharging test device. To date, considerable studies have been conducted on the thermoelectric properties of pure-phase cathode materials by electrochemical-calorimetry, whereas few studies have been performed on materials modified by coating. However, the high-temperature discharge and heat release have become serious with the rapid application of lithium-ion batteries, and the internal heat problem of high discharge rate batteries should be addressed. Therefore, the thermo-electrochemistry energy storage and thermostability of modified LiNi_0.8_Co_0.1_Mn_0.1_O_2_ cathode materials should be investigated.

In this study, LiNi_0.8_Co_0.1_Mn_0.1_O_2_ cathode material was coated with 3 wt.% Li_3_VO_4_, and the thermo-electrochemistry energy storage and thermostability of LiNi_0.8_Co_0.1_Mn_0.1_O_2_ cathode materials before and after modification were investigated using electrochemical-calorimetry combination technology. The combined thermodynamic and electrochemical methods in analyzing the LiNi_0.8_Co_0.1_Mn_0.1_O_2_ cathode material before and after modification is conducive for the optimisation of the thermal design and material development of the battery system and improvement of the safety performance of the battery. These conditions are of immense scientific significance to the research on high-performance LiNi_0.8_Co_0.1_Mn_0.1_O_2_ cathode materials.

## Experimental

### Materials preparation

The Ni_0.8_Co_0.1_Mn_0.1_(OH)_2_ precursor was mixed with LiOH·H_2_O at a molar ratio of 1:1.05, completely ground in an agate mortar and was calcined at a high temperature in an oxygen atmosphere to obtain the lithium-ion battery LiNi_0.8_Co_0.1_Mn_0.1_O_2_ cathode material. Then, a mechanical fusion method was used to add LiOH·H_2_O and V_2_O_5_ at a molar ratio of 3:1 to the ethanol solution for mixing and grinding. After the LiNi_0.8_Co_0.1_Mn_0.1_O_2_ cathode material was added for grinding, and the completely ground material was calcined at 700°C for 8 h. The Li_3_VO_4_-coated LiNi_0.8_Co_0.1_Mn_0.1_O_2_ cathode material was obtained.

### Battery assembly

The weights of positive electrode material, acetylene black and polyvinylidene fluoride were 0.3200, 0.0400 and 0.0400 g based on the mass ratio of 8:1:1. Then, in N-methyl-2-pyrrolidone solvent grinding for 20 min, the uniformity of viscous liquid was regularly coated on the aluminum foil and was placed in 80°C oven drying after 4 h to make a positive plate. Button batteries (type 2025) were assembled in a glove box filled with argon gas (99.999%) by using an anode shell, a cathode shell, a cathode piece, an anode piece (Li), a nickel net, a membrane (Celgard 2300), and an electrolyte [1 mol· L^−1^ LiPF_6_ (DMC+EMC+EC (volume ratio 1:1:1))]. Electrochemical cyclic voltammetry (CV) test were conducted on the CHI760E electrochemical system.

### Material analysis and characterization

The X-ray powder diffraction (XRD, Rint-2000, Rigaku) was analyzed to the phase structure of the pristine and Li_3_VO_4_-coated LiNi_0.8_Co_0.1_Mn_0.1_O_2_ samples. A scanning electron microscope (SEM, JEOL, JSM-5612LV) was used to analyse the microstructure of materials. The microstructure of the sample and surface coating of the material were observed by using a transmission electron microscope (HRTEM, Titan G2 60-300 with image corrector, 200 kV).

### Performance test of electrochemical-calorimetry combination

The thermo-electrochemical properties of LiNi_0.8_Co_0.1_Mn_0.1_O_2_ cathode materials before and after modification were analyzed by using LAND test system and TAM air calorimeter. The battery was placed in an ampoule that contains methyl silicone oil. The positive and negative electrodes of the battery were connected to a LAND test system by long copper wires and were placed in a calorimeter at 30, 40, and 50°C. The LAND test system has a charging and discharging voltage range of 2.5–4.3 V and a charge and discharge multiplier of 0.1, 0.5, 1, and 2°C in evaluating the battery's rate performance and cycle performance at different temperatures. The TAM air calorimeter is an eight-channel milliwatt-scale thermal conductivity isothermal calorimeter. The calorimeter is calibrated, the calibration constant is obtained, the experimental data are calibrated and the heat flow generated by the battery during charging and discharging is recorded.

## Results and discussion

### Morphology and interface analysis

**Figure 1:**
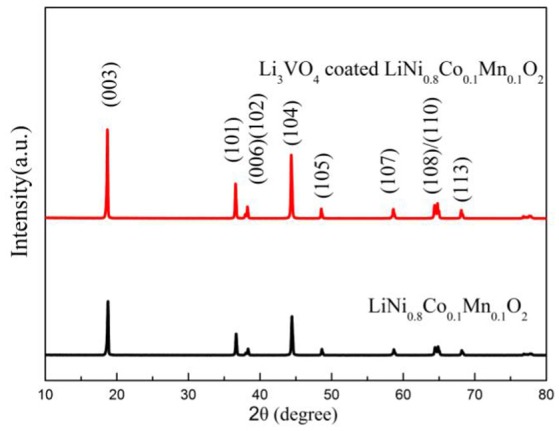
XRD patterns of LiNi_0.8_Co_0.1_Mn_0.1_O_2_ and Li_3_VO_4_-coated LiNi_0.8_Co_0.1_Mn_0.1_O_2_ cathode materials.

In order to study the effect of Li_3_VO_4_-coated LiNi_0.8_Co_0.1_Mn_0.1_O_2_ cathode materials, XRD was used to analyze the structure of LiNi_0.8_Co_0.1_Mn_0.1_O_2_ cathode materials before and after Li_3_VO_4_ coating, as shown in Figure 1. It can be seen from figure that the XRD pattern of the sample diffraction peak is consistent with the diffraction peak of R$\bar{3}$m space group and has the structure of α-NaFeO_2_. The diffraction peak of Li_3_VO_4_-coated LiNi_0.8_Co_0.1_Mn_0.1_O_2_ cathode material is quite sharp, indicating that the material has good crystallinity. The peak splitting of (108) and (110) is obvious, indicating that a better layered structure has been formed. According to the diffraction data, the I(003)/I(104) values of cathode materials were 1.265 (pristine), and 1.399 (coated). In cathode materials, I(003)/I(104)>1.2, indicating that the cation mixing degree of active materials is small. In conclusion, the Li_3_VO_4_-coated LiNi_0.8_Co_0.1_Mn_0.1_O_2_ cathode material will not change the layered phase or layered framework and can effectively inhibit the mixing degree of cation and improve the properties of the material.

**Figure 2:**
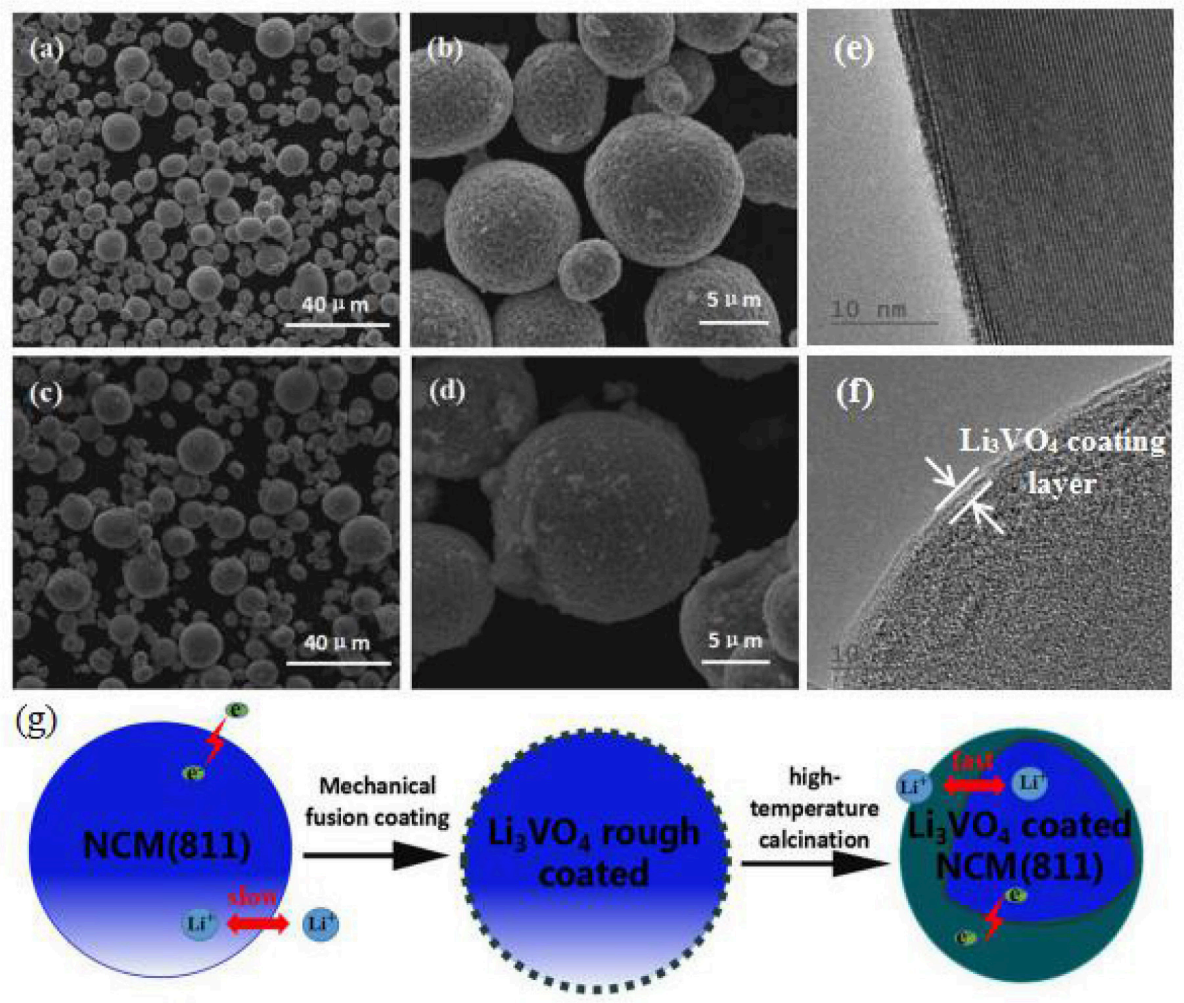
SEM images of LiNi_0.8_Co_0.1_Mn_0.1_O_2_ **(a,b)** and Li_3_VO_4_-coated LiNi_0.8_Co_0.1_Mn_0.1_O_2_ **(c,d)** cathode materials, TEM images of LiNi_0.8_Co_0.1_Mn_0.1_O_2_ **(e)**, and Li_3_VO_4_-coated LiNi_0.8_Co_0.1_Mn_0.1_O_2_ cathode material **(f)**, Schematic illustration **(g)** of the synthesis of Li_3_VO_4_-coated LiNi_0.8_Co_0.1_Mn_0.1_O_2_ cathode materials.

**Figure 3:**
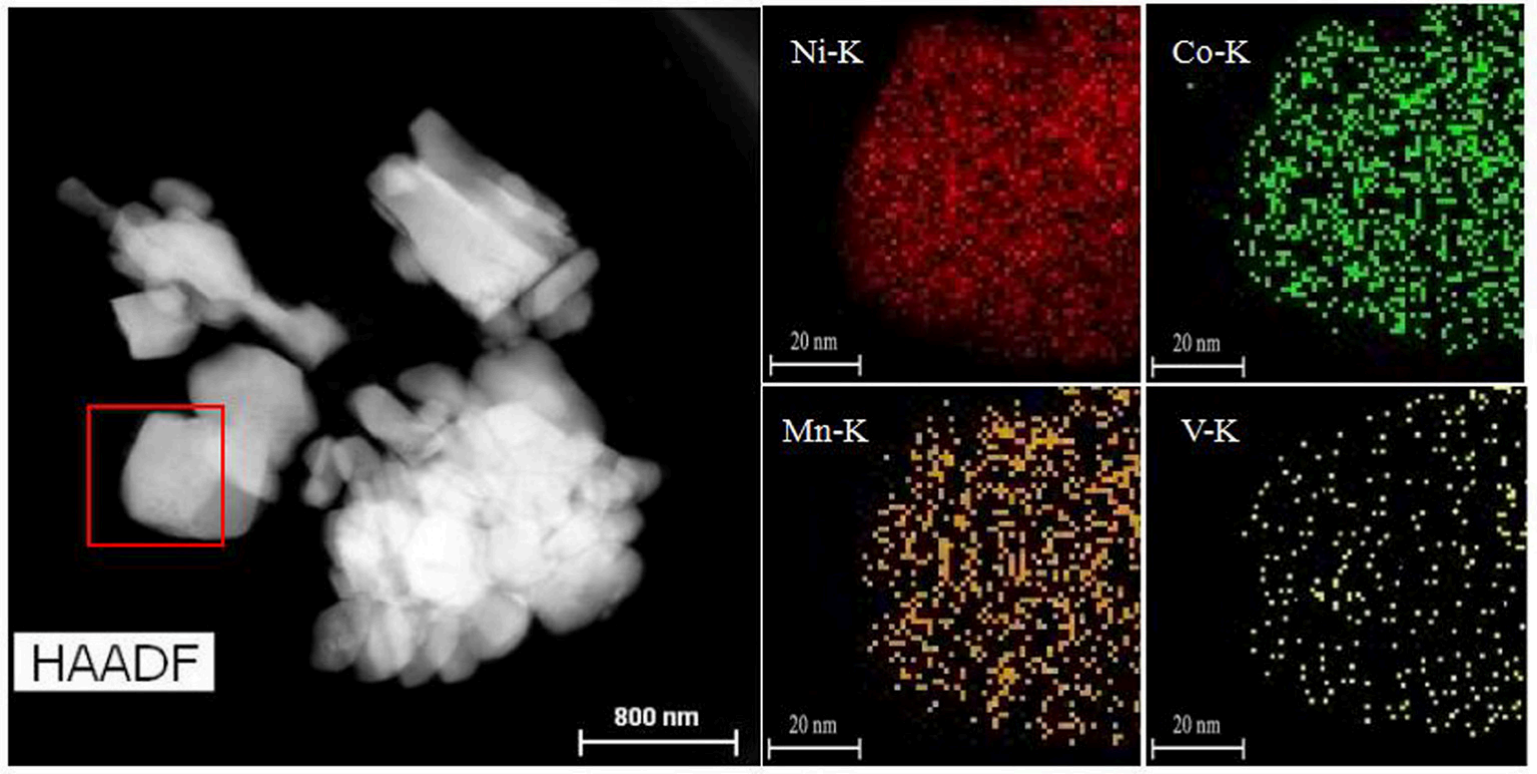
HAADF-STEM images of Li_3_VO_4_-coated LiNi_0.8_Co_0.1_Mn_0.1_O_2_ cathode materials and Mapping of Nickel, Cobalt, Manganese, and Vanadium elements.

The SEM images of all samples are illustrated in Figures 2a–d, which is mainly used to analyse and evaluate the surface morphology of the sample. As shown in Figures 2a–d, LiNi_0.8_Co_0.1_Mn_0.1_O_2_ and Li_3_VO_4_-coated LiNi_0.8_Co_0.1_Mn_0.1_O_2_ cathode materials have spheroid structure, and their spheroid size is approximately 8 μm. Li_3_VO_4_ can be filled tightly on LiNi_0.8_Co_0.1_Mn_0.1_O_2_ particles because spheroid particles have good fluidity. Figures 2c,d show that the shape and size of the LiNi_0.8_Co_0.1_Mn_0.1_O_2_ material after coating slightly change, and the Li_3_VO_4_-coated surface becomes rough, which is the sample close between particles and particles. This condition may be due to the melted Li_3_VO_4_ gathered in the modification process of heat treatment. In addition, we conducted TEM analysis of LiNi_0.8_Co_0.1_Mn_0.1_O_2_ and Li_3_VO_4_-coated LiNi_0.8_Co_0.1_Mn_0.1_O_2_, as shown in Figures 2e,f. As can be seen from the Figure 2f, there is a clear Li_3_VO_4_ layer on the surface of the coated sample, and the thickness of the coating is about 1~2 nm, which indicates that Li_3_VO_4_ is successfully coated on the surface of LiNi_0.8_Co_0.1_Mn_0.1_O_2_. The schematic illustration of the synthesis of Li_3_VO_4_-coated LiNi_0.8_Co_0.1_Mn_0.1_O_2_ cathode material is shown in Figure 2g. A mechanical fusion method is applied in the LiNi_0.8_Co_0.1_Mn_0.1_O_2_ ethanol soluble solution mixed with LiOH·H_2_O and V_2_O_5_ grinding, and the Li_3_VO_4_-coated LiNi_0.8_Co_0.1_Mn_0.1_O_2_ cathode material is obtained under 700°C calcination. To study the degree of material coating, we further analyse the Li_3_VO_4_-coated LiNi_0.8_Co_0.1_Mn_0.1_O_2_ HAADF-STEM of cathode materials and the mapping diagram of Ni, Co, Mn, and V elements, as shown in Figure 3. The element mapping signal is obtained by scanning the HAADF- rectangle in the STEM. As shown in Figure 3, Ni, Co, Mn, and V elements are uniformly distributed in the material, which indicate that Li_3_VO_4_ is uniformly coated on the surface of LiNi_0.8_Co_0.1_Mn_0.1_O_2_ cathode material. In Li_3_VO_4_-coated LiNi_0.8_Co_0.1_Mn_0.1_O_2_ cathode material, Li_3_VO_4_ acts as a fast ion conductor layer that promotes lithium ion transport, reduces the electrolyte between the active material and side effects and improves the thermal stability and electrochemical performance of materials.

### Electrochemical properties

**Figure 4:**
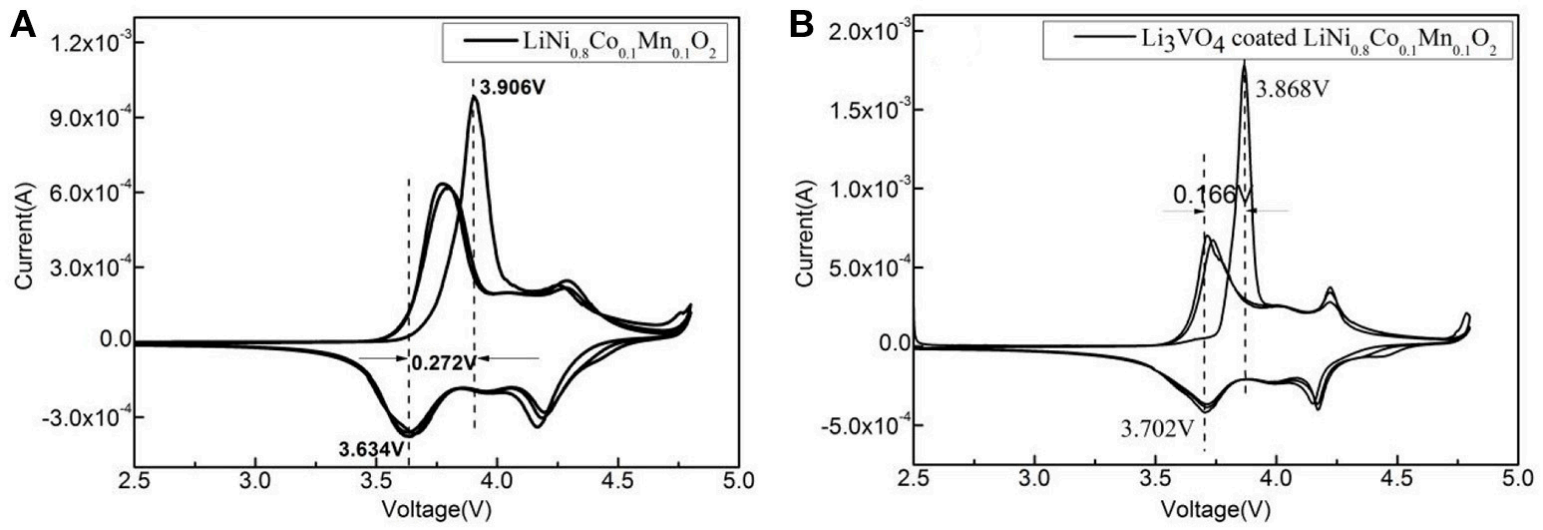
Cyclic voltammetry curve of LiNi_0.8_Co_0.1_Mn_0.1_O_2_ **(A)** and Li_3_VO_4_-coated LiNi_0.8_Co_0.1_Mn_0.1_O_2_ cathode material **(B)**.

Figure 4 shows the cyclic voltammetry curve of LiNi_0.8_Co_0.1_Mn_0.1_O_2_ and Li_3_VO_4_-coated LiNi_0.8_Co_0.1_Mn_0.1_O_2_. It can be seen from Figure 4 that all the peaks are similar and no significant new redox peak appears after Li_3_VO_4_ coating, indicating that the modification by Li_3_VO_4_ does not change the main structure of LiNi_0.8_Co_0.1_Mn_0.1_O_2_. The potential difference of the redox peak of Li_3_VO_4_-coated LiNi_0.8_Co_0.1_Mn_0.1_O_2_ is 0.166 V and the pure LiNi_0.8_Co_0.1_Mn_0.1_O_2_ sample is 0.272 V, which indicated Li_3_VO_4_-coated LiNi_0.8_Co_0.1_Mn_0.1_O_2_ has smaller potential difference. The larger the potential difference between the deimmobilization and embedding of lithium ions, the greater the electrode polarization effect. It indicating that the Li_3_VO_4_ surface layer inhibits the direct contact between the active material and the electrolyte, enhances the lithium ion diffusion between the electrode/electrolyte interface, and improves the electrochemical performance of the battery. At the same time, the peak area of the cyclic voltammetry curve was integrated. It was found that after Li_3_VO_4_-coated LiNi_0.8_Co_0.1_Mn_0.1_O_2_, the peak area of the first cycle were closer to the second and third cycles, indicating that Li_3_VO_4_-coated LiNi_0.8_Co_0.1_Mn_0.1_O_2_ cathode material has better reversibility. Compared with pure LiNi_0.8_Co_0.1_Mn_0.1_O_2_, Li_3_VO_4_-coated LiNi_0.8_Co_0.1_Mn_0.1_O_2_ cathode material has better lithium diffusion kinetics.

**Figure 5:**
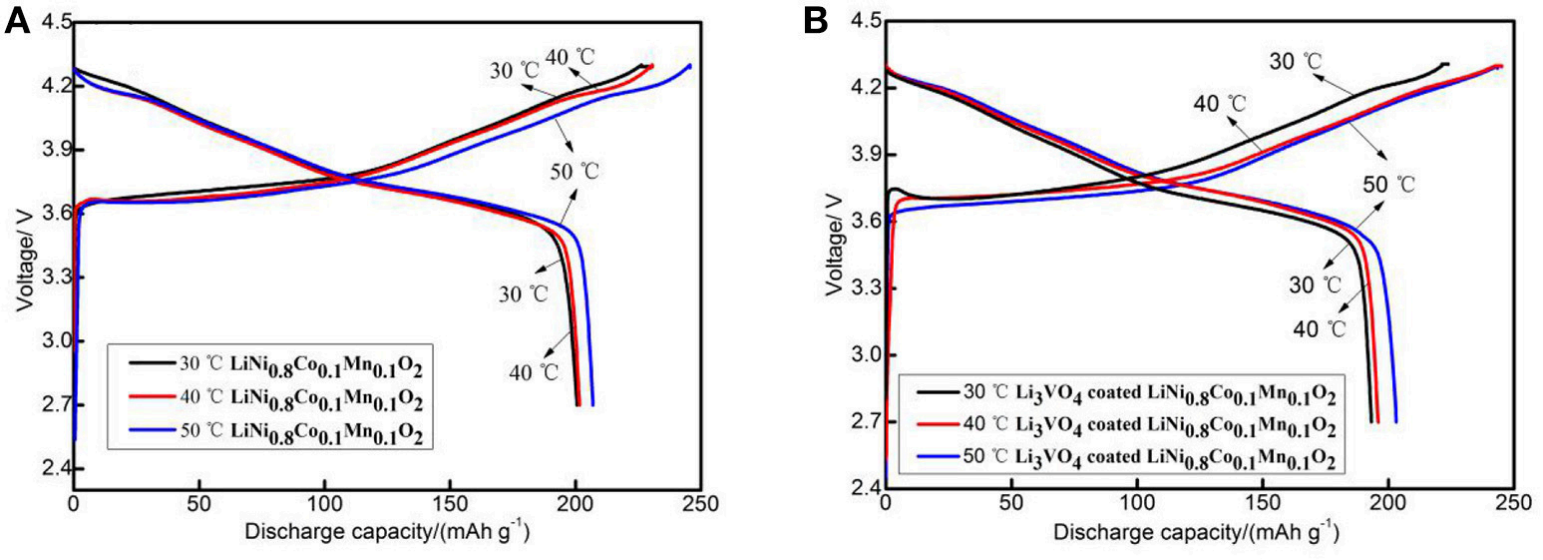
The initial charge-discharge curves of LiNi_0.8_Co_0.1_Mn_0.1_O_2_ **(A)** and Li_3_VO_4_-coated LiNi_0.8_Co_0.1_Mn_0.1_O_2_ **(B)** cathode materials at different temperatures.

**Figure 6:**
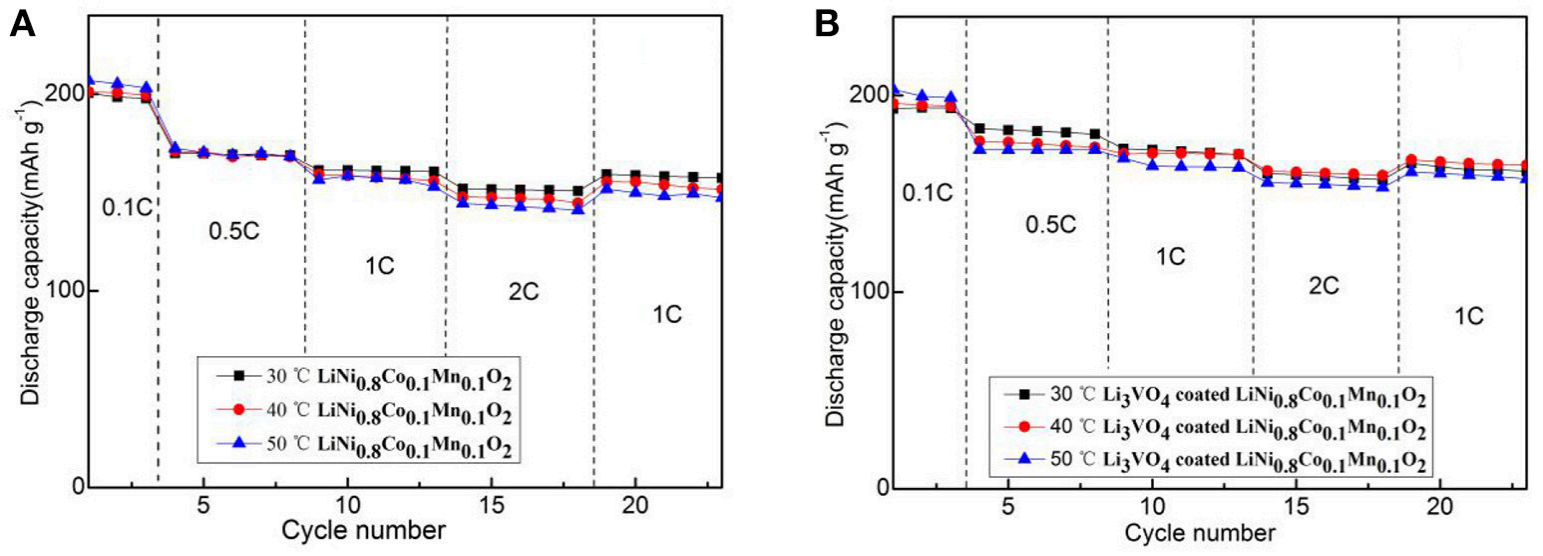
The rate performance of LiNi_0.8_Co_0.1_Mn_0.1_O_2_ **(A)** and Li_3_VO_4_-coated LiNi_0.8_Co_0.1_Mn_0.1_O_2_ **(B)** cathode materials at different temperatures and rates.

Figure 5 depicts the initial charge/discharge capacities curve of the pristine and Li_3_VO_4_-coated LiNi_0.8_Co_0.1_Mn_0.1_O_2_ cathode material at 0.1°C. All the samples show initial sloping region and smooth charge/discharge curves with similar capacities. The initial discharge capacities of pristine samples were 200.6 (30°C), 201.6 (40°C) and 207.0 mAh g^−1^(50°C). The initial discharge capacities of Li_3_VO_4_-coated LiNi_0.8_Co_0.1_Mn_0.1_O_2_ cathode materials were 193.2 (30°C), 195.9 (40°C), and 203.1 mAh g^−1^(50°C). The higher the ambient temperature, the more adequate the initial reaction of the battery, so the initial discharge capacity increases slightly as the temperature increases. Figure 6 shows the rate performance of LiNi_0.8_Co_0.1_Mn_0.1_O_2_ and Li_3_VO_4_-coated LiNi_0.8_Co_0.1_Mn_0.1_O_2_ cathode materials under different rates (0.1, 0.5, 1, and 2°C) at 30, 40, and 50°C. As shown in the figure, the discharge capacity decreases with increased current density in the charging and discharging processes. The lithium-ion battery shows a high initial capacity when the temperature increases from 30 to 50°C. However, the cycle stability of the battery decreases and capacity decay rate increases. It can be seen from the figure that LiNi_0.8_Co_0.1_Mn_0.1_O_2_ has the best rate performance at 30°C, and Li_3_VO_4_-coated LiNi_0.8_Co_0.1_Mn_0.1_O_2_ has the best rate performance at 40°C, indicating that Li_3_VO_4_-coated LiNi_0.8_Co_0.1_Mn_0.1_O_2_ cathode material is more suitable at high temperatures. The increase in electrode polarization during charging is the main cause for the rapid decline of battery capacity at high temperature. The discharge specific capacity is similar to the specific discharge capacity at 1°C for the first time when the material is charged and discharged after returning to 1°C at 0.1, 0.5, 1, and 2°C, which indicates that the Li_3_VO_4_-coated cathode materials does not affect the reversibility of LiNi_0.8_Co_0.1_Mn_0.1_O_2_ cathode material. In addition, the temperature increases, and the discharge capacity of Li_3_VO_4_-coated LiNi_0.8_Co_0.1_Mn_0.1_O_2_ cathode materials is remarkably better than that of pure LiNi_0.8_Co_0.1_Mn_0.1_O_2_ with increased current density. This condition is because the reaction inside the battery accelerates and the side effects increase with increased temperature. However, in Li_3_VO_4_-coated LiNi_0.8_Co_0.1_Mn_0.1_O_2_ cathode material, Li_3_VO_4_ promotes lithium ion transport, reduces the high-temperature electrolyte between the active material and side effects and improves the stability of materials under high-temperature conditions. Therefore, the Li_3_VO_4_-coated LiNi_0.8_Co_0.1_Mn_0.1_O_2_ cathode material has the best rate performance.

**Figure 7:**
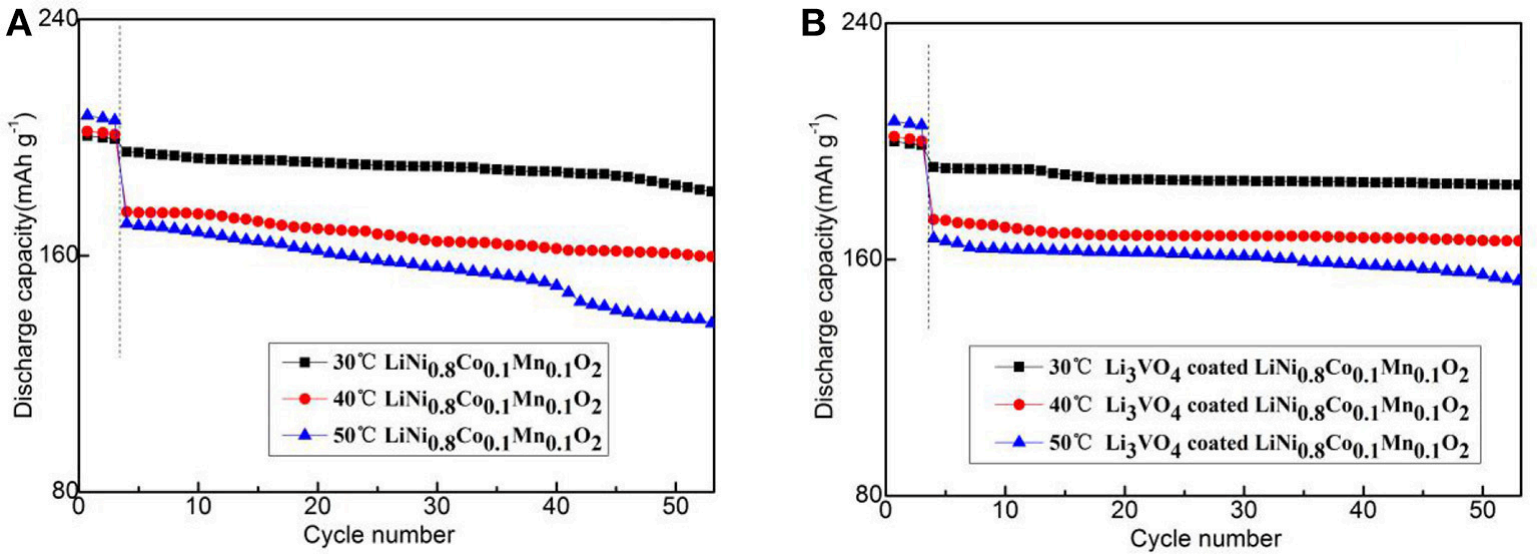
Cycle performance of LiNi_0.8_Co_0.1_Mn_0.1_O_2_ **(A)** and Li_3_VO_4_-coated LiNi_0.8_Co_0.1_Mn_0.1_O_2_ **(B)** cathode materials at different temperatures under 1°C.

Figure 7 shows the cycle performance of LiNi_0.8_Co_0.1_Mn_0.1_O_2_ and Li_3_VO_4_-coated LiNi_0.8_Co_0.1_Mn_0.1_O_2_ cathode material under different temperatures (30, 40, and 50°C) at 1°C for 50 cycle times. As shown in figure, the Li_3_VO_4_-coated LiNi_0.8_Co_0.1_Mn_0.1_O_2_ cathode material exhibit an excellent cycle stability after three cycles of activation at 0.1°C. The sharp reduction in the discharge capacities after three cycles is caused by the changes in the current density and temperature. The specific discharge capacities of LiNi_0.8_Co_0.1_Mn_0.1_O_2_ cathode materials at 30, 40, and 50°C at 1°C are 197.1, 176.8, and 172.8 mAh·g^−1^, respectively. The capacity retention ratios are 93.2, 91.4, and 80.4% after 50 charge/discharge cycles. For the Li_3_VO_4_-coated LiNi_0.8_Co_0.1_Mn_0.1_O_2_ cathode material, the specific discharge capacities are 194.2 (30°C), 175.5 (40°C), and 168.1 (50°C) mAh·g^−1^ after 50 times of charge/discharge cycle capacity retention of 96.7, 95.9, and 91.4%, respectively. With the increase of temperature, the decay rate of LiNi_0.8_Co_0.1_Mn_0.1_O_2_ is large, while the Li_3_VO_4_-coated LiNi_0.8_Co_0.1_Mn_0.1_O_2_ cathode material is relatively stable. This condition is because the lithium ion diffusion of pure LiNi_0.8_Co_0.1_Mn_0.1_O_2_ cathode material is relatively slow and cannot maintain with the electron transfer rate. Thus, electrode electrochemical polarization occurs, which causes capacity loss and performance degradation. In addition, passive film formation is accelerated in the cycle process by accelerating the oxidation electrolyte decomposition of LiNi_0.8_Co_0.1_Mn_0.1_O_2_ between the cathode materials and electrolyte and the adverse event that occurs between the binder and electrolyte. The thick organic passivation film on the surface of cathode material particles leads to increased interface impedance of anode/solution, which decreases the battery's high-temperature circulating capacity. After the modification of LiNi_0.8_Co_0.1_Mn_0.1_O_2_ by Li_3_VO_4_ coating, the diffusion rate of lithium ions is accelerated and the interface impedance of the positive/solution is reduced, which is consistent with the analysis results of rate performance. Therefore, the capacity retention rate of LiNi_0.8_Co_0.1_Mn_0.1_O_2_ cathode material coated with Li_3_VO_4_ is remarkably improved with excellent cycling performance.

### Thermoelectric chemical properties

**Figure 8:**
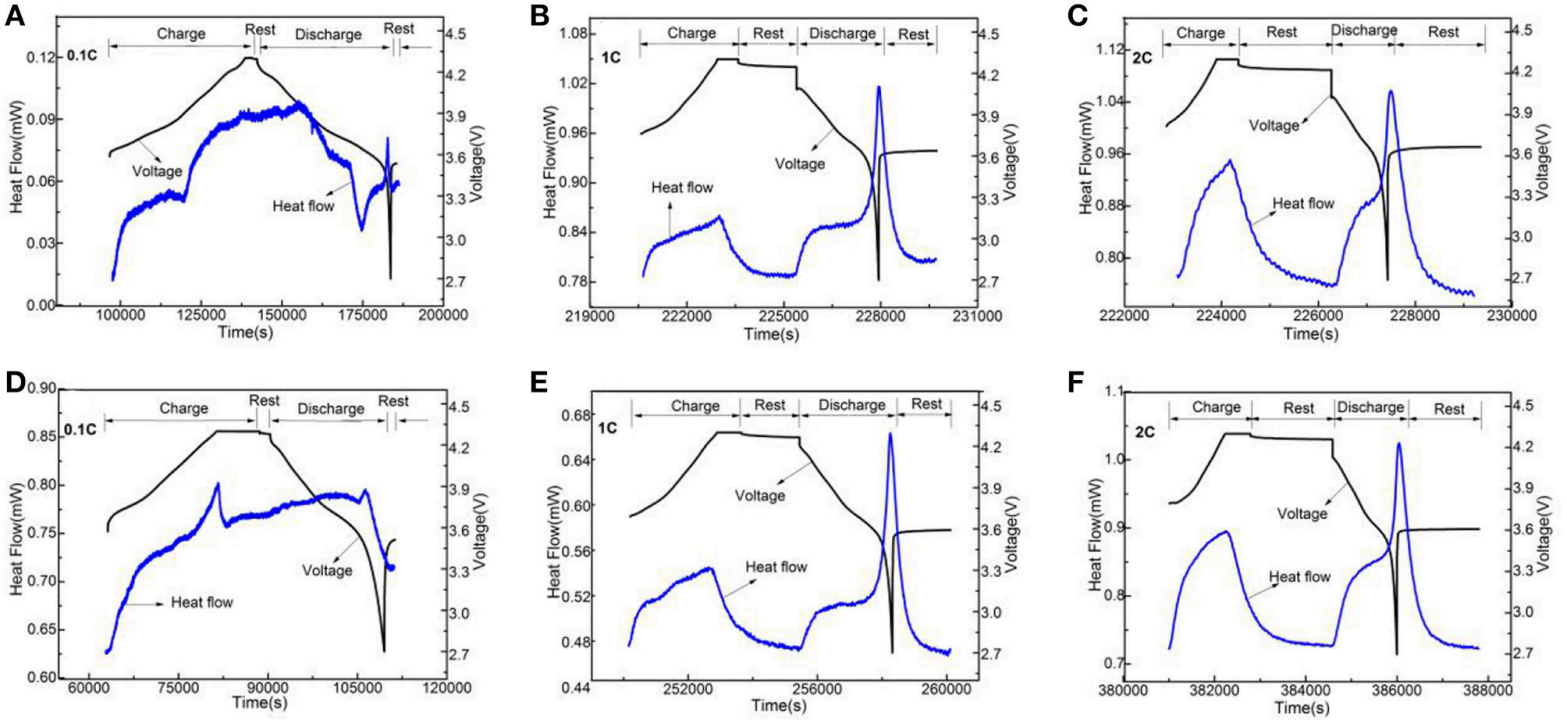
The change curves of heat flow and voltage of LiNi_0.8_Co_0.1_Mn_0.1_O_2_ **(A–C)** and the Li_3_VO_4_-coated LiNi_0.8_Co_0.1_Mn_0.1_O_2_ **(D**−**F)** cathode materials with time under 30°C at different rates.

**Figure 9:**
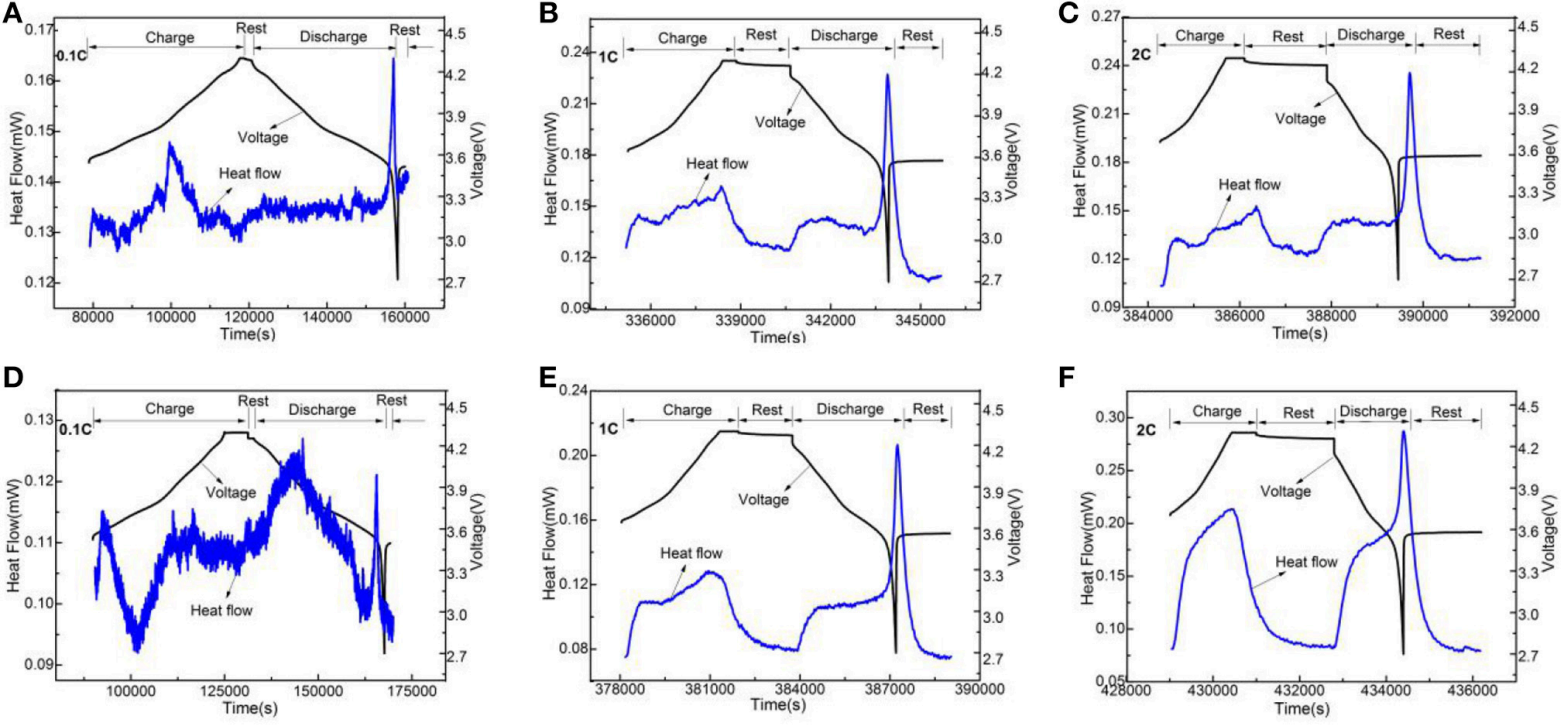
The change curves of heat flow and voltage of LiNi_0.8_Co_0.1_Mn_0.1_O_2_ **(A–C)** and the Li_3_VO_4_-coated LiNi_0.8_Co_0.1_Mn_0.1_O_2_ **(D–F)** cathode materials with time under 40°C at different rates.

**Figure 10:**
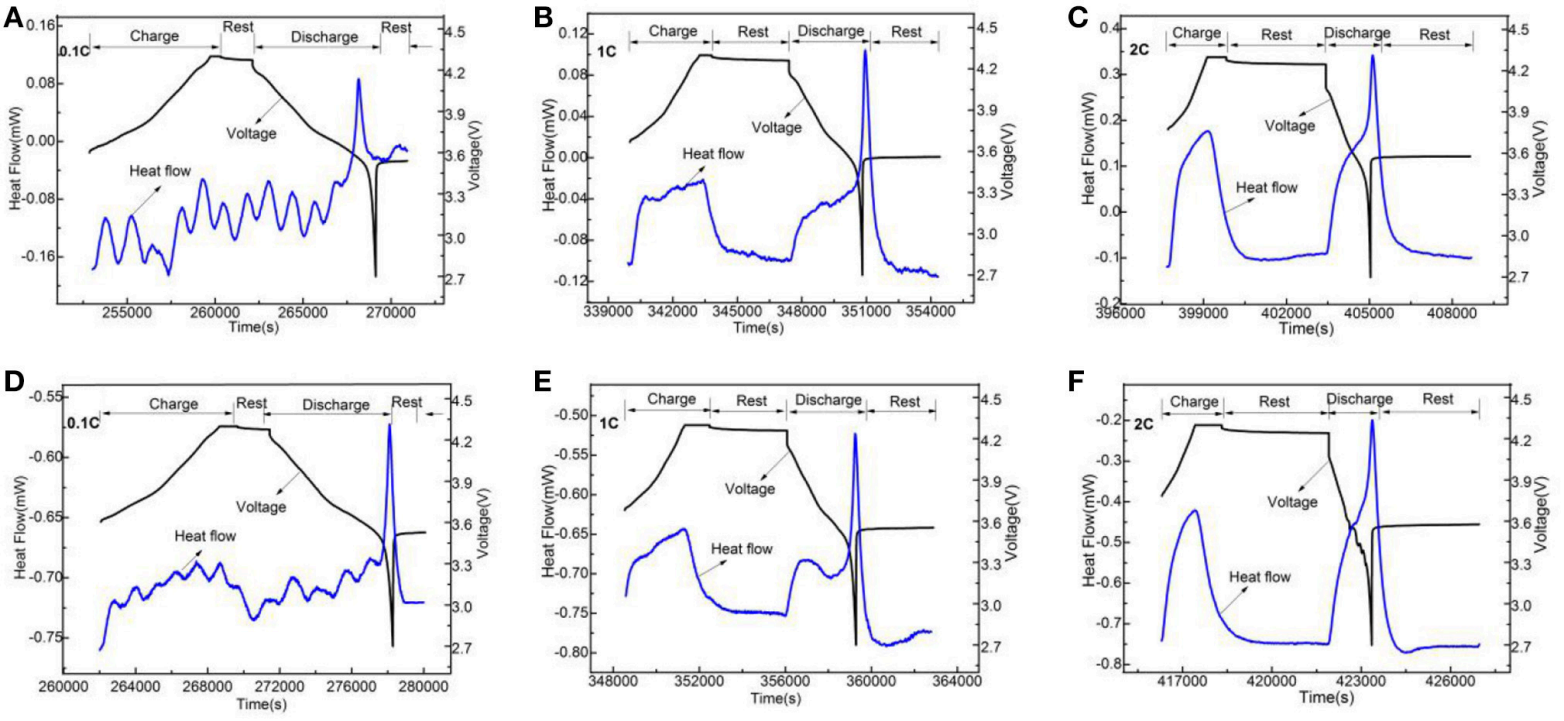
The change curves of heat flow and voltage of LiNi_0.8_Co_0.1_Mn_0.1_O_2_ **(A–C)** and the Li_3_VO_4_-coated LiNi_0.8_Co_0.1_Mn_0.1_O_2_ **(D–F)** cathode materials with time under 50°C at different rates.

In order to analyze Li_3_VO_4_-coated LiNi_0.8_Co_0.1_Mn_0.1_O_2_ cathode material further, we used electrochemical calorimetry to analyze the thermo-electrochemical properties. Figures 8–10 show the heat-time and voltage-time curves of LiNi_0.8_Co_0.1_Mn_0.1_O_2_ and the Li_3_VO_4_-coated LiNi_0.8_Co_0.1_Mn_0.1_O_2_ cathode material at different temperatures (30, 40, and 50°C) and different rates (0.1, 1, and 2°C). As shown in the heat flow time curve in Figures 8–10, the heat flow curve of the cathode material in the charging and discharging processes shows many exothermic peaks with the change of time under the low rate of 0.1°C. However, the heat flow curve shows obvious exothermic peak and no impurity peak under 1 and 2°C. This condition is because the battery can be approximated as a reversible process during charge and discharge and the reversible heat and irreversible heat are relatively close in at a low rate. In this case, the exothermic peak under 1 and 2°C is generated by the superposition of electrode polarization and battery chemical reaction. However, the heat generated by electrode polarization is dominant. In general, the polarization heat of the battery gradually increases with increased magnification. Thus, the polarization heat exceeds the chemical reaction heat of the battery, and the peak of reaction heat can be completely covered. Therefore, the exothermic peak under high rate is generated by the superposition of electrode polarization and battery chemical reaction, and the heat generated by electrode polarization is dominant. To determine the thermal electrochemical properties of cathode materials before and after coating, the electric quantity and heat generated during the charging and discharging processes of the battery are obtained by integrating the current-time and heat flow-time curves. The formula is expressed as follows:

(1) $$\begin{array}{rcl} {\text{Q}}_{\text{ch}} & = & \overset{t_{1}}{\underset{0}{\int }}\text{i}\left(t\right)dt, \end{array}$$

(2) $$\begin{array}{rcl} {\text{Q}}_{\text{disch}} & = & \overset{t_{1}}{\underset{0}{\int }}\text{i}\left(t\right)dt, \end{array}$$

(3) $$\begin{array}{rcl} {\text{Q}}_{\text{total}} & = & {\text{Q}}_{ch}+{\text{Q}}_{disch}, \end{array}$$

(4) $$\begin{array}{rcl} {\text{q}}_{\text{ch}} & = & \overset{t_{1}}{\underset{0}{\int }}h\left(t\right)dt, \end{array}$$

(5) $$\begin{array}{rcl} {\text{q}}_{\text{disch}} & = & \overset{t_{2}}{\underset{0}{\int }}h\left(t\right)dt, \end{array}$$

(6) $$\begin{array}{rcl} {\text{q}}_{\text{total}} & = & {\text{q}}_{ch}+{\text{q}}_{disch}, \end{array}$$

(7) $$\begin{array}{rcl} \Delta q & = & q_{ch}-q_{disch}, \end{array}$$

where Q_total_ represents the amount of electricity consumed during charge (Q_ch_) and discharge (Q_disch_) of the battery, expressed in C, i(t) represents the change of current with time, expressed in mA, t is the time of the entire charge and discharge processes of the battery, expressed in s, q_total_ is the charge (q_ch_), and discharge (q_disch_) of the battery heat production with time, Δq is the difference between the amount of charge generated by the battery and the amount of discharge, expressed in mJ, and h(t) represents the change value of heat flow with time, expressed in mW. On the basis of the total amount of electricity consumed by the battery during charging and discharging, the total number of moles of reaction can be calculated. The formula is expressed as follows:

(8) $$\begin{array}{r} \text{n}=\frac{Q}{F}. \end{array}$$

where n is the total number of moles of the reaction, F is Faraday's constant and its value is 96 485°C·mol^−1^. The total enthalpy change (Δ_r_H_m_) of chemical reaction can be calculated based on the relationship between the total heat produced by the battery during charging and discharging and the total number of moles of reaction, which is expressed in kJ·mol^−1^. Entropy (Δ_r_S_m_) is a measure of the disorder degree of the reaction system, which is expressed in J·mol^−1^·K^−1^. The formula is expressed as follows:

(9) $$\begin{array}{rcl} \Delta_{r}H_{m} & = & \frac{q}{n}, \end{array}$$

(10) $$\begin{array}{rcl} \Delta_{r}{\text{S}}_{\text{m}} & = & -\frac{\Delta \text{q}}{2\text{nT}}=-\frac{{\text{q}}_{\text{ch}}-{\text{q}}_{\text{disch}}}{2\text{nT}}. \end{array}$$

As shown in Table 1, the heat yield of lithium ion battery cathode material increases when the temperature increases from 30 to 50°C. This condition is because the temperature inside the battery increases, and the redox reaction speed increases with increased temperature. At this time, the side reaction accompanying the battery is intense, and the reaction heat of the battery side is increased, which increases the total heat of the battery. The heat production of the material before and after Li_3_VO_4_ coating is similar at 30 and 40°C. However, the heat yield of Li_3_VO_4_-coated LiNi_0.8_Co_0.1_Mn_0.1_O_2_ cathode material under 50°C at 1 and 2°C is less than LiNi_0.8_Co_0.1_Mn_0.1_O_2_. This condition is due to the high-temperature coating layer that protects the material from electrolyte erosion and reduces the side effects between the electrolyte and cathode material, which reduces the battery heat yield and improves the thermal stability and security of materials.

**Table 1:** Thermodynamic parameters for LiNi_0.8_Co_0.1_Mn_0.1_O_2_ and Li_3_VO_4_-coated LiNi_0.8_Co_0.1_Mn_0.1_O_2_ cathode materials.

**Temperatue (^°^C)**	**Rate (C)**	**q_total_ (mJ)**	**Δq (mJ)**	**Q_total_ (C)**	**q_total_ (mJ)**	**Δq (mJ)**	**Q_total_ (C)**
		**LiNi**_**0.8**_**Co**_**0.1**_**Mn**_**0.1**_**O**_**2**_			**Li**_**3**_**VO**_**4**_**-coated LiNi**_**0.8**_**Co**_**0.1**_**Mn**_**0.1**_**O**_**2**_		
	0.1	−35.86	2.70	2.71	−31.68	2.16	2.42
30	1	−215.18	11.46	1.59	−203.05	6.95	1.71
	2	−297.52	19.66	1.09	−289.34	12.88	1.32
	0.1	−86.10	19.7	2.43	−84.11	6.49	2.62
40	1	−299.74	29.08	1.88	−290.00	22.68	2.05
	2	−438.53	37.81	1.34	−429.87	32.97	1.70
	0.1	−123.46	32.10	2.61	−118.83	12.81	2.81
50	1	−598.54	47.46	2.22	−480.94	32.14	1.85
	2	−1008.94	54.54	1.478	−908.23	51.21	1.63

“*–” stands for exothermic*.

**Figure 11:**
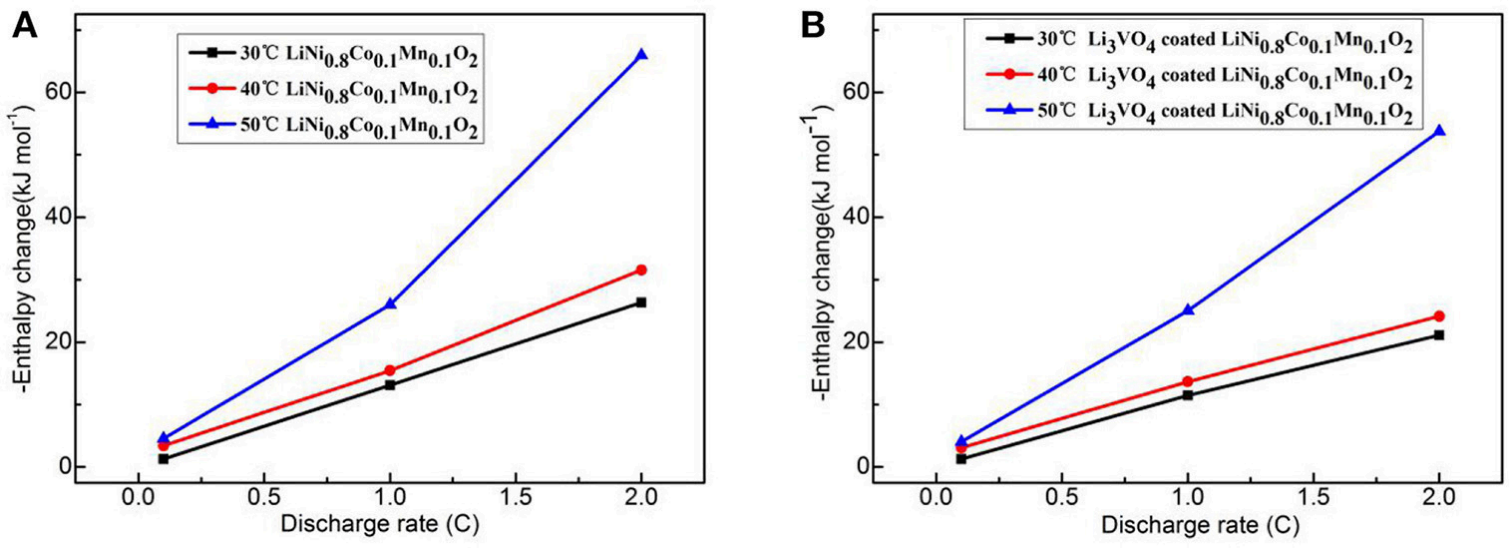
Curve of enthalpy change with rate for LiNi_0.8_Co_0.1_Mn_0.1_O_2_ **(A)** and Li_3_VO_4_-coated LiNi_0.8_Co_0.1_Mn_0.1_O_2_ **(B)** cathode materials at different temperatures.

Figure 11 shows the change curve of enthalpy of LiNi_0.8_Co_0.1_Mn_0.1_O_2_ cathode materials with current rate at different temperatures before and after Li_3_VO_4_ coating. At the same temperature, the enthalpy increases with increased rate. At the same current rate, the enthalpy increases with increased temperature. The greater the change in enthalpy is, the worse safety condition of the battery will be. The difference between the Li_3_VO_4_-coated LiNi_0.8_Co_0.1_Mn_0.1_O_2_ and pure LiNi_0.8_Co_0.1_Mn_0.1_O_2_ cathode material is 12.19 kJ·mol^−1^ when the temperature is 50°C and the charge–discharge rate is 2°C. The Li_3_VO_4_-coated LiNi_0.8_Co_0.1_Mn_0.1_O_2_ cathode material has a low enthalpy change, which indicates that the thermal stability of the material is high and secure at high temperatures.

**Figure 12:**
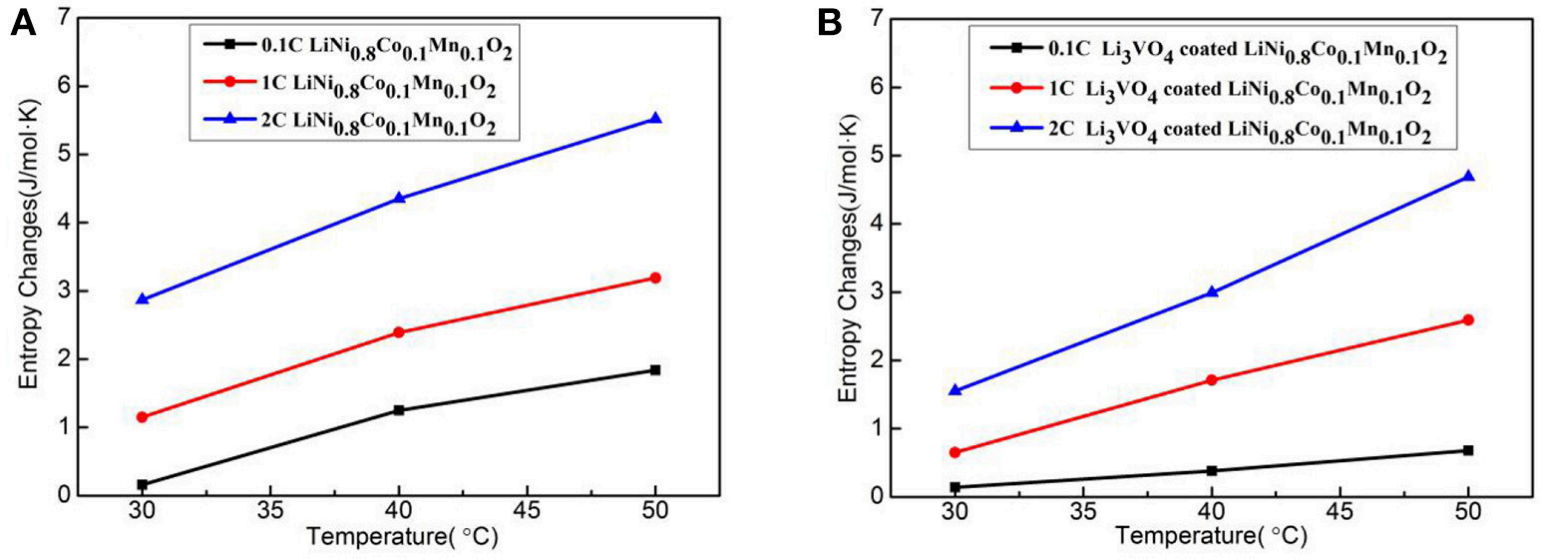
Curve of entropy change with temperature for LiNi_0.8_Co_0.1_Mn_0.1_O_2_ **(A)** and Li_3_VO_4_-coated LiNi_0.8_Co_0.1_Mn_0.1_O_2_ **(B)** cathode materials at different rates.

Figure 12 shows the entropy change with temperature for LiNi_0.8_Co_0.1_Mn_0.1_O_2_ (Figure 12A) and Li_3_VO_4_-coated LiNi_0.8_Co_0.1_Mn_0.1_O_2_ (Figure 12B) cathode materials at different rates. In the reaction system, the entropy increases with increased temperature and rate. The second law of thermodynamics states that any change or chemical reaction in an isolated system is constantly in the direction of an increase in entropy. Under this principle, the principle of entropy increase is applicable when a battery system and its surrounding environment are considered as a new isolated system. The battery reaction rate increases with increased temperature at 0.1, 1, and 2°C. However, the entropy value of Li_3_VO_4_-coated LiNi_0.8_Co_0.1_Mn_0.1_O_2_ cathode material is less than that of pure LiNi_0.8_Co_0.1_Mn_0.1_O_2_, which indicates that the Li_3_VO_4_-coated LiNi_0.8_Co_0.1_Mn_0.1_O_2_ cathode material is more orderly in the charge–discharge reaction process and the side reaction between the active material and electrolysis is less with higher structure stability.

## Conclusions

The energy storage and thermostability properties of the LiNi_0.8_Co_0.1_Mn_0.1_O_2_ cathode materials before and after Li_3_VO_4_ coating at different temperatures and rate were investigated by electrochemical calorimetry for the first time. The results showed that the specific discharge capacities of Li_3_VO_4_-coated LiNi_0.8_Co_0.1_Mn_0.1_O_2_ cathode material at 1°C are 194.2 (30°C), 175.5 (40°C), and 168.1 (50°C) mAh g^−1^ and the capacity retention rates are 96.7, 95.9, and 91.4%, with excellent rate and cycle performance after 50 charge/discharge cycles. Thermo-electrochemical experiments indicated that the current rate increase, heat quantity, enthalpy and entropy increase, and the specific discharge capacity decreases in the charge and discharge processes of lithium-ion battery cathode materials at the same temperature. The temperature increase, heat quantity, enthalpy and entropy increase and the specific discharge capacity increases first and rapidly decays at the same current rate. In addition, the heat production, enthalpy change, and entropy change of the Li_3_VO_4_-coated LiNi_0.8_Co_0.1_Mn_0.1_O_2_ cathode material in the charge–discharge reaction system are lower than that of pure LiNi_0.8_Co_0.1_Mn_0.1_O_2_ at 30, 40, and 50°C. This finding shows that Li_3_VO_4_ coating remarkably improves the thermal stability of the material. This condition is because the Li_3_VO_4_ coating layer as a fast ion conductor promotes lithium ion transport, protects the active material from electrolyte erosion, reduces the side effects and electrode polarization and improves the thermal stability of the Li_3_VO_4_-coated LiNi_0.8_Co_0.1_Mn_0.1_O_2_ cathode materials, which have excellent electrochemical performance and thermal stability. In conclusion, Li_3_VO_4_-coated LiNi_0.8_Co_0.1_Mn_0.1_O_2_ cathode materials is proven to be an effective method in improving the energy storage and thermostability properties of cathode materials. This experiment will provide a new direction and basis for the study on high-temperature and thermo-electrochemical properties of lithium ion batteries.

## Author contributions

LS and FT conceived the idea. FT and LS prepared all materials. LS, FT, and ZX conducted electrochemical-calorimetry experiments. LS, FT, and ZX analyzed the data. FT wrote the manuscript. LS, ZC, and HZ commented on it. LS, ZX, ZC, and HZ supervised the implementation of the project.

### Conflict of interest statement

The authors declare that the research was conducted in the absence of any commercial or financial relationships that could be construed as a potential conflict of interest.
